# Nanoparticles loaded with natural medicines for the treatment of Alzheimer’s disease

**DOI:** 10.3389/fnins.2023.1112435

**Published:** 2023-10-09

**Authors:** Nanyang Liu, Juanjuan Ruan, Hao Li, Jianhua Fu

**Affiliations:** ^1^Xiyuan Hospital, China Academy of Chinese Medical Sciences, Beijing, China; ^2^Department of Geriatrics, Zhumadian Hospital of Traditional Chinese Medicine, Zhumadian, Henan Province, China; ^3^Wangjing Hospital, China Academy of Chinese Medical Sciences, Beijing, China

**Keywords:** Alzheimer’s disease, nanoparticles, natural medicines, Chinese herbal medicines, blood–brain barrier

## Abstract

Alzheimer’s disease (AD) is a progressive neurodegenerative disease that disrupts cognitive function and severely affects the quality of life. Existing drugs only improve cognitive function and provide temporary relief of symptoms but do not stop or delay disease progression. Recently, natural medicines, especially Chinese herbal medicines, have gained attention in the treatment of AD due to their antioxidant, anti-inflammatory, and neuroprotective effects. However, conventional oral dosage forms lack brain specificity and have side effects that lead to poor patient compliance. Utilizing nanomedicine is a promising approach to improve brain specificity, bioavailability, and patient compliance. This review evaluates recent advances in the treatment of AD with nanoparticles containing various natural medicines. This review highlights that nanoparticles containing natural medicines are a promising strategy for the treatment of AD. It is believed that this technology can be translated into the clinic, thereby providing opportunities for AD patients to participate in social activities.

## 1. Introduction

Alzheimer’s disease (AD), the most common type of dementia, is a degenerative disease of the central nervous system. Patients often have a variety of symptoms, mainly memory loss, which seriously damage the quality of life and health of the elderly (Villemagne et al., 2018). The pathogenesis of AD is still unclear, and the hallmark pathology of the disease is the deposition of amyloid β (Aβ) and phosphorylated tau nerve fibers (Congdon and Sigurdsson, 2018). The latest published data show that in 2019, there were approximately 57.4 million people with dementia in the world, and this number is expected to increase to 150 million in 2050 (Collaborators, 2022). Existing drugs are far from meeting the treatment expectations of this large population of patients. Traditional therapeutic strategies, including acetylcholinesterase inhibitors, are often limited due to insufficient solubility, reduced bioavailability, and the presence of another side effect. Moreover, FDA-approved drugs for the treatment of AD include tacrine, rivastigmine, galantamine, donepezil, and memantine, and the use of tacrine is limited due to associated side effects, including liver toxicity (Wilson and Geetha, 2020). Therefore, other therapeutic strategies need to be explored urgently.

Recently, natural medicines, especially Chinese herbal medicines, which act on AD through multiple targets, have been regarded as promising therapeutic approaches (Zhang C. et al., 2019; Lye et al., 2021). The blood–brain barrier (BBB) is an important biological barrier between the circulatory and central nervous systems. While blocking toxins, the BBB can also prevent many drugs from reaching the nervous system. It has been reported that 98% of small-molecule drugs cannot cross the BBB and act on the nervous system (Neumaier et al., 2021; Smith-Cohn et al., 2022). Moreover, the processing mechanisms for natural medicine make it difficult for the drug to pass through the BBB, and these drugs have low solubility in brain tissue (Zhang J. et al., 2019). Patients usually need to increase the dose to achieve adequate plasma concentrations. However, high doses may cause damage due to toxicity (Ma et al., 2019; Anwar et al., 2021; Zhang et al., 2021b).

In recent years, with the advancement of nanotechnology, researchers have found that drugs loaded into nanoparticles can be transported across the BBB to the diseased site, increasing the concentration of drugs in the nervous system, which has great prospects in improving drug utilization. In this article, we review the recent progress in the use of nanomaterials in AD treatment, as well as the key roles of several natural compounds, especially Chinese herbal medicine-loaded nanoparticles, which may provide more opportunities for the development of future neurodegenerative disease treatments.

## 2. The challenge of natural drug delivery—BBB

The BBB refers to the barrier between plasma and brain cells formed by capillary walls and glial cells and the barrier between plasma and cerebrospinal fluid formed by the choroid plexus. It is a basic framework composed of vascular endothelial cells interconnected by tight junction proteins and adhesion junction proteins (Han, 2021). The BBB can restrict the entry of peripheral macromolecular proteins, cytotoxic substances, and peripheral immune cells into the nerve center, thereby maintaining the stability of the internal environment of the central nervous system (Yang et al., 2021). Oxygen, carbon dioxide, and smaller lipophilic molecules easily pass through the BBB (Han and Jiang, 2021). However, some macromolecular substances are not able to pass through. A major problem in treating AD is the presence of BBB, which does not allow many drugs to enter the brain in sufficient concentrations (Whelan et al., 2021). Treatment is further hindered by unwanted systemic side effects due to drug release from the carrier before reaching the brain. Although the BBB does not allow many drugs that can be used to treat AD to enter the brain, many targeted strategies have been investigated to deliver drugs through the BBB. Nanoparticles are one of the most promising approaches.

## 3. Role of nanoparticles in AD pharmacotherapy

Nanoparticle drug delivery systems such as liposomes, chitosan nanoparticles, magnetic nanoparticles, and polymer micelles can be used as effective delivery vehicles for a variety of drugs such as anticancer drugs, antiviral drugs, antibiotics, peptides and proteins, nucleic acids, vaccines, and diagnostic drugs (Huete-Carrasco and Lavelle, 2023). According to the target organ, different delivery methods can be designed, such as injection, oral administration, nasal administration, ocular administration, brain delivery, and DNA gene nano-delivery systems.

More and more studies have shown that nanosizing changes the pharmacodynamics and pharmacokinetics of drugs *in vivo*. Nanoparticle drug delivery systems can enhance the efficacy of drugs while reducing adverse reactions (Fang et al., 2023); increase the solubility of drugs, improve adhesion and increase surface area to improve absorption and increase bioavailability (Wu et al., 2023); passively target drugs to organs such as the liver, spleen, lung, bone marrow and lymph nodes or actively target them after modification to change the distribution of drugs *in vivo* and increase drug concentration at target sites; regulate the circulation time of drugs *in vivo* and control the release rate of drug molecules to achieve sustained-release effects and prolong the duration of drug action; prevent biotech drugs, vaccines, nucleotides, and other substances from being enzymatically deactivated *in vivo* to protect them (Hirschbiegel et al., 2023). In addition, it can also improve the stability of protein and peptide drugs in the digestive tract to establish new routes of administration. Because nanoparticle drug delivery systems can endow drug formulations with many new features, their efficacy in AD treatment is gradually being recognized (Sahni et al., 2011; Doggui et al., 2012; Zhu et al., 2021). Nanoparticles can effectively deliver drugs to cells to treat AD and reduce Aβ aggregation and tau hyperphosphorylation (Abbas, 2021). This technology improves the delivery of drugs to the brain *via* nasal administration by enhancing the adhesion with nasal mucosa, protecting the encapsulated drugs from biodegradation, and promoting the extracellular transport of P-GP efflux protein (Battaglia et al., 2018; Mota et al., 2023). This not only works on symptoms but also prevents progressive neuronal loss (Satapathy et al., 2021). More importantly, the treatment of AD patients can be improved to a greater extent by enhancing the BBB permeability (Zheng et al., 2017).

**Figure 1:**
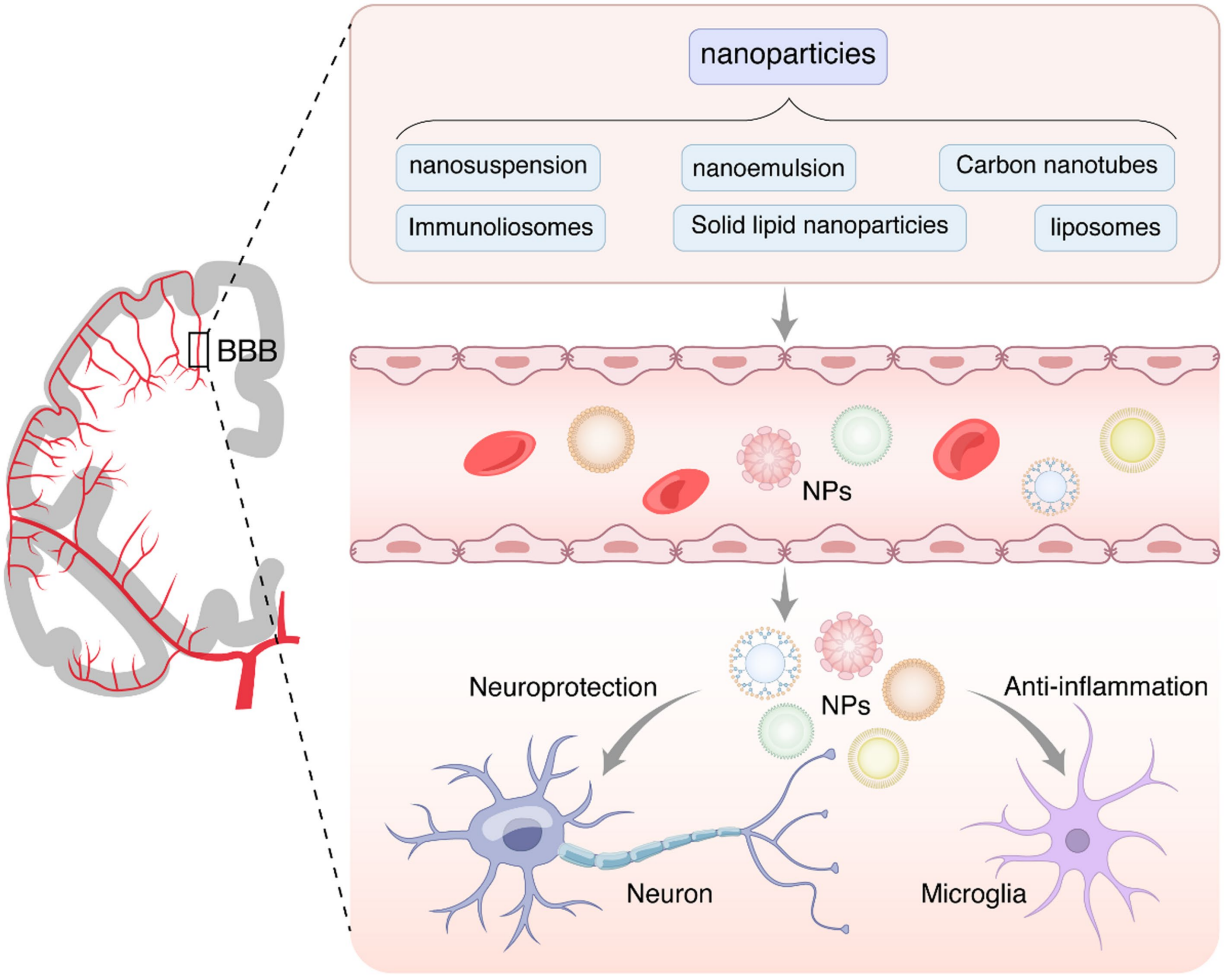
The nanoparticles penetrate the blood–brain barrier and act on the central nervous system. The presence of the BBB is crucial for preventing harmful substances from entering the brain from the bloodstream. However, it also hinders the transfer of most small-molecule drugs and large molecules (such as peptides, proteins, and gene-based drugs), severely limiting the treatment of central nervous system diseases, such as neurodegenerative diseases, brain tumors, brain infections, and strokes. Nanoparticles, such as nanosuspensions, nanoemulsions, carbon nanotubes, immune liposomes, solid lipid nanoparticles, and liposomes, can be administered through the gastrointestinal system, respiratory tract, and nasal cavity. They can effectively traverse the blood–brain barrier and enter target brain cells, including neurons, microglial cells, and astrocytes. Natural compounds often face challenges in penetrating the blood–brain barrier. Therefore, nanoparticles can be employed to facilitate the delivery of active ingredients from natural compounds to the central nervous system, thereby exerting neuroprotective, anti-inflammatory, and antioxidant effects.

The pathways through which nanotechnologies cross the BBB mainly include carrier-mediated transport, receptor-mediated transport, adsorption-mediated transport, and cell-mediated transport (Tang et al., 2019), making it easier for them to cross the BBB and gain access to target brain cells, including neurons, microglia, and astrocytes, for neuroprotective and anti-inflammatory effects (Figure 1). In short, the use of nanotechnology could enable lower drug concentrations in blood, better therapeutic efficacy, and lower drug toxicity and could provide more options and strategies for AD drug development (Fu et al., 2019; Tsolou et al., 2020; Zhang W. et al., 2021).

## 4. Nanoparticle-loaded natural medicines for AD

**Figure 2:**
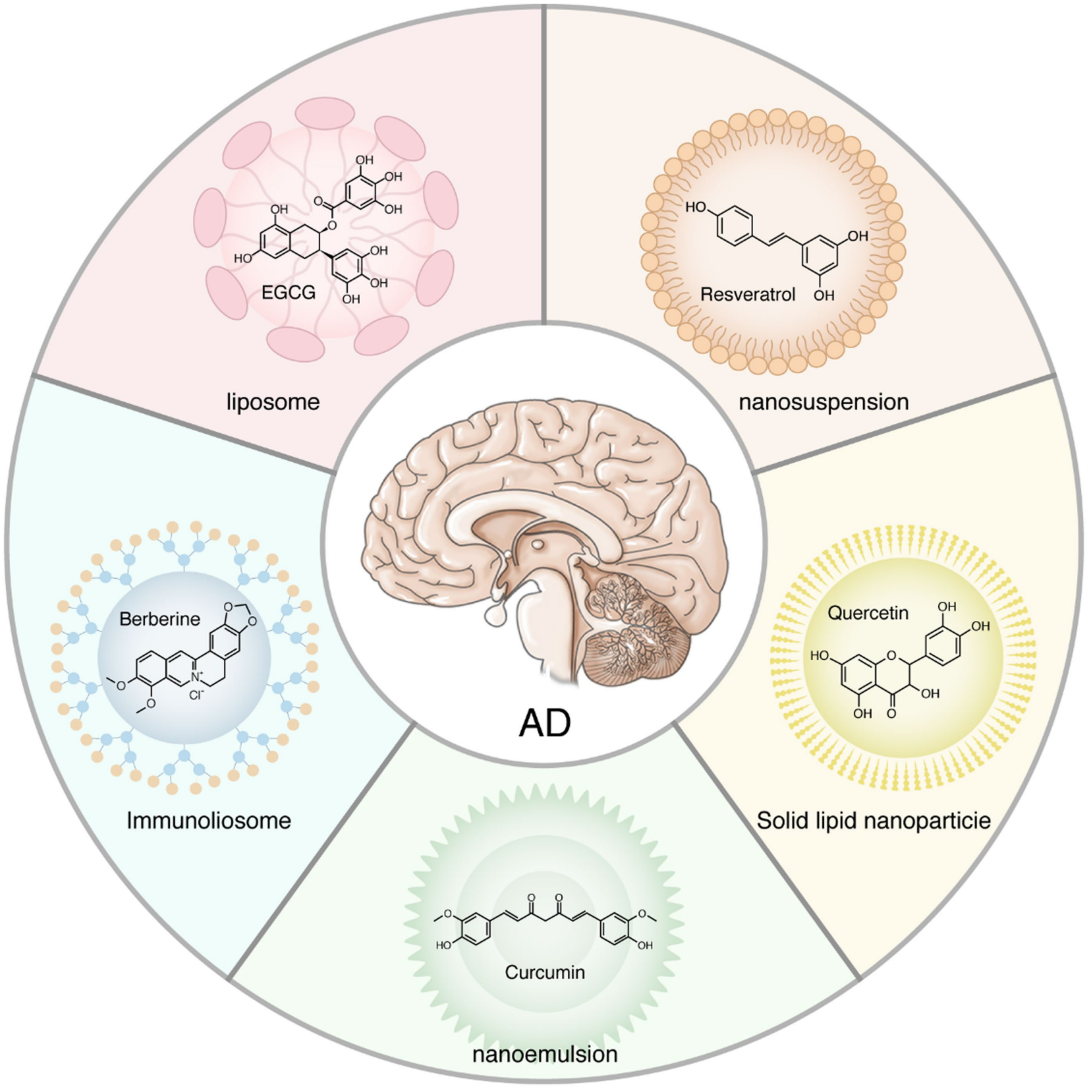
Natural herbal extract loaded on the nanoparticles. Natural medicines, especially Chinese herbal medicines, including but not limited to curcumin, berberine, quercetin, resveratrol, epigallocatechin gallate, and triptolide, are widely used to treat Alzheimer’s disease. However, these extracts tend to have large molecular weights that make it difficult for them to penetrate the blood–brain barrier to reach the brain. Herbal extracts loaded on the nanoparticles can maximize their utilization.

At present, there are approximately 35,000 kinds of natural herbs in the world that can be used as medicinal sources. The extracts or active ingredients of these natural medicines (such as flavonoids, polyphenols, terpenes, etc.) are beneficial to human health. The functions of these ingredients are very broad, with antipsychotic, antifatigue, antioxidant, antidepressant, anxiolytic, anti-inflammatory, antitumor, and other effects (Zhang C. et al., 2019). In particular, herbs that exhibit antidepressant, anti-inflammatory, and antioxidant properties may be beneficial for the treatment of AD patients. However, the disadvantages of these drugs, such as large molecular weight, poor solubility, and toxicity, limit their clinical use. An optimal combination of nanotechnology and natural extracts could overcome this limitation. Here, we describe several of the most commonly used AD-targeted herbal extracts, including but not limited to curcumin, berberine, quercetin, resveratrol, epigallocatechin gallate, and triptolide. They are loaded on different nanoparticles, such as nanosuspension, nanoemulsion, carbon nanotubes, immunoliosomes, solid lipid nanoparticles, and liposomes, and then administered through the gastrointestinal system, respiratory tract, or nasal cavity in a way that more easily permeates the BBB to the central nervous system (Figure 2).

### 4.1. Curcumin

Curcumin, a hydrophobic polyphenol extracted from the rhizome of turmeric, is widely used in Asia to treat inflammatory diseases due to its remarkable anti-inflammatory properties (Abdulmalek et al., 2021). It is also widely used as a dietary spice in daily life. This compound has been introduced as an ideal candidate for the treatment of different neurological diseases because of its ability to cross the BBB. Despite the pleiotropic activity of curcumin, its poor solubility, fast clearance, and low stability limit its clinical applications (Barbara et al., 2017). Therefore, many nanoformulation-based strategies have been pursued over the past few decades to improve its properties in the preclinical setting. Types of nanoformulations include lipids/liposomes, water/micro/nanogels, micelles, and nanoparticles (Abbas, 2021). Encapsulation of curcumin in nanoparticles has been shown to improve chemical stability and prevent enzymatic and pH-mediated degradation. In addition, loading curcumin into nanoparticles increased its circulation in the body (Abbas, 2021).

The main pathological features of AD include extracellular amyloid plaques and intracellular neurofibrillary tangles (Goyal et al., 2021). Although current hypotheses emphasize different mechanisms, such as Aβ deposition, tau hyperphosphorylation, oxidative stress, neuroinflammation, and altered cholinergic and glutamatergic neurotransmission, the Aβ cascade and tau aggregation remain the most widely accepted central factors that trigger and/or accelerate AD pathogenesis. Numerous experimental reports have demonstrated the potential role of curcumin in AD treatment by illustrating its ability to reduce inflammation, activate neurogenesis, and inhibit Aβ production (Zhang H. et al., 2021). Epidemiological studies have highlighted the association between type 2 diabetes mellitus and AD. Targeting different pathways in the brain with type 2 diabetes mellitus therapy provides a novel and attractive strategy to treat diabetes-related neuronal changes. Abdulmalek et al. (2021) injected male Wistar rats intraperitoneally with streptozotocin to generate a diabetes model. The expression of amyloid-related genes, levels of Aβ accumulation, and levels of tau hyperphosphorylation were significantly reduced by feeding the rats curcumin nanoparticles for 6 months. The findings are consistent with those of two other studies (Gao et al., 2020; Noor et al., 2022). The nanoparticles carry T807 molecules attached to the surface of the red blood cell membrane. With the suitable physicochemical properties of PLGA nanoparticles and the unique biological function of the red blood cell membrane, RPCNPs stabilize and promote the sustained release of curcumin, thereby increasing its time in circulation. This formulation demonstrates enhanced BBB penetration and a high binding affinity for hyperphosphorylated tau in neural cells, inhibiting multiple key pathways in the pathogenesis of tau-related AD. Huo et al. (2019) also developed a novel curcumin nano-delivery method using PLGA nanoparticles. They hypothesized that curcumin bioaction and drug delivery properties could improve bioavailability by altering the PLGA surface and selenium nanoparticle encapsulation. As expected, the curcumin-loaded Se-PLGA nano delivery system reduced Aβ loading in AD mouse brain samples, greatly reversing memory deficits. Furthermore, the specific binding of curcumin-loaded Se-PLGA nanospheres to Aβ plaques was observed by fluorescence microscopy. More importantly, the antioxidant capacity of curcumin-loaded PLGA nanoparticles against peroxy radicals was higher than that of free curcumin and the control. Overall, these two studies provide evidence that PLGA nanoparticles are a promising tool for AD drug delivery. Zhang et al. (2020) compared two curcumin nanocarrier formulations, namely, curcumin-encapsulated chitosan-coated polylactide-glycolide nanoparticles and curcumin-encapsulated hydroxypropyl-β-cyclodextrin complexes. Both formulations reduced the cytotoxicity of curcumin and showed comparable antioxidant effects. While the BBB protects the brain from attack by macromolecular particles, it also hinders the entry of therapeutic drugs. Improving BBB penetration is a critical step in the treatment of neurological disorders. Nanostructured lipid carriers (NLCs), solid colloidal drug delivery systems made by encapsulating or sandwiching the drug in a lipid core, offer one possibility. This approach is widely used as a delivery vehicle for drug nanotechnology due to its low toxicity, high biocompatibility, and biodegradability. Sadegh Malvajerd et al. (2019) encapsulated curcumin in NLCs to increase drug accumulation in the brain. Pharmacokinetic studies showed that the amount of curcumin available was significantly higher in the brains of subjects treated with curcumin-loaded NLCs than in the brains of those treated with free curcumin. The preparation process did not have any significant effect on the antioxidant activity of curcumin. Another study using NLCs found that curcumin nanoformulations can effectively cross the BBB and promote the sustained release of curcumin (Meng et al., 2015). Taken together, these two studies suggest that NLCs are beneficial for BBB penetration and drug release. The above studies demonstrate that nanoformulations of curcumin significantly enhance its efficacy and bioavailability under *in vitro* and *in vivo* conditions. Among them, NLCs are more suitable for BBB penetration. Various nanoparticle technologies have been developed for the loading of curcumin, suitable for reducing the dose of the main therapeutic agent, which can improve the therapeutic effect while reducing systemic toxicity.

### 4.2. Quercetin

Quercetin, a flavonoid found primarily in fruits and vegetables, such as onions, asparagus, red leaf lettuce, and apples, has been shown to protect neurons from oxidative damage while reducing lipid peroxidation (Pinheiro et al., 2020a). It also contributes to the clearance of Aβ, counteracting cell lysis and inhibiting the inflammatory cascade. Despite broad pharmacological properties, its low solubility, poor absorption, and rapid metabolism limit its clinical applications (Pinheiro et al., 2020b). In addition, quercetin aglycone is less effective after crossing the BBB. Therefore, clinical studies of quercetin and analogs as neuroprotective agents must aim to improve their bioavailability, which may improve clinical efficacy. Various strategies have been attempted to address these issues, such as developing molecules with greater BBB penetration and selecting nanoparticle technologies.

Studies have found that the dysfunction of metal ions in the brain may be related to the etiology of AD (Liu et al., 2019). Disrupting these metal-peptide interactions using nanoparticles holds considerable promise as a therapeutic strategy against this incurable disease. In this context, Sun et al. (2016) employed PLGA-functionalized quercetin (PLGA@QT) nanoparticles to investigate the effect on Aβ fibrils. As expected, PLGA@QT NPs inhibited Zn^2+^-induced neurotoxicity and enhanced neuronal cell viability. Most encouragingly, the *in vivo* systemic toxicity of PLGA@QT NPS detected by histological analysis of major organs did not show any signs of adverse effects in mice. Similarly, Rifaai et al. (2020) investigated the neuroprotective effect of quercetin nanoparticles in an aluminum chloride-induced AD model. Administration of quercetin nanoparticles significantly reduced neuronal degeneration, senile plaques, and neurofibrillary tangles. Furthermore, the investigators encapsulated quercetin in NLCs functionalized with transferrin and noted that the formulated particles attempted to cross the BBB *via* transferrin receptors overexpressed in brain endothelial cells. Cytotoxicity assays performed in the hCMEC/D3 cell line showed that even the highest concentration of nanoparticles induced no cytotoxicity after 4 h of incubation. This delivery strategy allowed for increased quercetin penetration into the BBB and significantly inhibited Aβ fibril formation. Pinheiro et al. (2020b) employed RVG29 peptide-functionalized NLCs systemically loaded with quercetin to target nicotinic acetylcholine receptors. The permeability of RVG29 nanoparticles through the BBB in the *in vitro* model was significantly increased by 1.5-fold achieved using nonfunctionalized nanoparticles after 4 h of incubation with the cells. Recently, some nanobubbles with diameters of only tens of micrometers have attracted attention. Liu et al. (2020) formed the Qc@SNPs-MB system by embedding quercetin-modified sulfur nanoparticles (Qc@SNPs) into microbubbles (MB). The system is combined with focused ultrasound to instantaneously open the BBB. The rapid accumulation of Qc@SNPs in the brain effectively reduces neuronal apoptosis, the inflammatory response, calcium homeostasis imbalance, and oxidative stress mediated by endoplasmic reticulum stress and protects nerve cells, thereby achieving the goal of treating AD.

### 4.3. Resveratrol

Polyphenols in natural products are widely used to prevent age-related diseases due to their anti-inflammatory and neuroprotective effects. Among the known polyphenols, resveratrol (RES) is widely found in fruits, especially grapes, berries, and nuts. RES has many therapeutic activities, including anti-inflammatory, antioxidant, antiproliferative, and neuroprotective activities (Guan et al., 2016; Labban et al., 2021). Given these efficacies and the ability to bind to Aβ_1-42_, it has been considered a candidate for AD treatment. Oral administration is traditionally the most preferred route of administration. However, RES suffers from many disadvantages associated with its oral use, including low solubility, poor bioavailability, chemical instability, hepatic metabolism, and rapid elimination (Komorowska et al., 2020). Encapsulation of RES into nanocarriers can overcome the barriers of instability and solubility, leading to improved bioavailability. These strategies include solid lipid nanoparticles, selenium nanoparticles, lipid core nanocapsules, transfer bodies, and nanoemulsions.

Resveratrol encapsulated into selenium nanoparticles (RSV-SeNPs) attenuates oxidative stress and mitochondrial dysfunction. Additionally, it increases Aβ clearance by ameliorating cholinergic deficits (Abozaid et al., 2022). Salem et al. (2019) developed two resveratrol-loaded transfer bodies and nanoemulsions, both of which can maximize access to the BBB. The transporter has higher BBB permeability than that of the nanoemulsion and provides significantly more improvement in the spatial memory of amnestic rats than that achieved with the nanoemulsion. As previously mentioned, selenium nanoparticles have unique Aβ absorption properties. In this context, Yang et al. (2018) loaded resveratrol into selenium nanoparticles to construct Res@SeNP structures. This structure leads to significantly more BBB penetration than that achieved using pure drugs. More importantly, PC12 cells can be protected from Aβ^42^-Cu^2+^ complex-induced cell death. Previous studies have shown that intravenously administered resveratrol is rapidly metabolized to glucuronic acid and sulfate conjugates, which are eliminated in less than 2 h. Antibody-functionalized solid lipid nanoparticles transport resveratrol to the brain and prolong its metabolism *in vivo* (Loureiro et al., 2017). The above *in vivo* and *in vitro* studies showed that nanobody-encapsulated resveratrol could effectively penetrate the BBB and was then observed to be abundantly enriched in the brain, which could improve its bioavailability and clinical efficacy.

### 4.4. Epigallocatechin gallate

Epigallocatechin gallate (EGCG) is a polyphenol extracted from green tea plants with strong antioxidant activity. It is a safe molecule that has shown therapeutic efficacy in the treatment of numerous diseases, such as Down syndrome, cancer, and neurological disorders (Granja et al., 2016, 2017). Especially in AD, it can inhibit tau aggregation, reduce Aβ accumulation, and inhibit inflammatory factor expression at different steps. However, EGCG has disadvantages in its medicinal chemistry and a high degree of instability in particular (Krupkova et al., 2016). One strategy to reduce drug clearance is to load drugs into nanostructured systems. It was found that dual drug-loaded PEGylated PLGA nanoparticles (EGCG/AA NPs) exhibited higher stability (Salem et al., 2019). Oral treatment of APP/PS1 transgenic mice with EGCG/AA NPs resulted in a significant increase in synapses and reduced neuroinflammation as well as Aβ plaque burden (Cano et al., 2019). Various animal studies have shown that prolonged exposure to aluminum causes neuropathological changes that impair the learning ability of rats (Prema et al., 2017). In addition, aluminum supplementation causes neurodegeneration and apoptotic neuronal loss as well as cognitive dysfunction (Granja et al., 2016). Drugs targeting metal aluminum ions may be useful for AD treatment. Epigallocatechin-gallate-loaded nanoparticles exhibited significant neuroprotective effects in aluminum chloride-induced AD model mice (Singh et al., 2018). In addition, the synthesized EGCG-stabilized selenium nanoparticles can effectively inhibit Aβ fibrillation and disintegrate preformed Aβ fibrils into nontoxic aggregates (Zhang et al., 2014).

### 4.5. Berberine

Berberine is a protoberberine alkaloid with an isoquinoline structure that can be extracted from traditional Chinese medicines such as Coptis Rhizoma, Cortex Phellodendri, and Sangean. Pharmacological studies suggest that berberine has a variety of pharmacological effects, such as regulation of blood sugar and blood lipids and anti-inflammatory and antiviral effects (Živančević et al., 2022). In addition, berberine can not only improve the spatial learning and memory of AD model rats but also inhibit the aggregation of Aβ and tau hyperphosphorylation and is one of the potential drugs for the prevention and treatment of AD (Fang et al., 2020; Raju et al., 2021). Selective penetration of the BBB limits its clinical use. Brain-targeted lipid-coated mesoporous silica nanoparticles (MSNs-BBR-L) with berberine had an eightfold higher BBB permeability than that of oral administration of pure berberine in feed (Singh et al., 2021). Overall, this study highlights the utility of MSNs-BBR-L as a promising drug delivery vehicle for targeting the brain. Zhang et al. (2021a) prepared berberine-HSA nanoparticles by the desolvation method and evaluated the interaction of HSA with berberine by molecular docking. Studies highlight that berberine interacts with HSA spontaneously through electrostatic interactions. Furthermore, pretreatment of neuronal cultures with berberine-HSA nanoparticles reduced H_2_O_2_-stimulated cytotoxicity and associated morphological changes.

### 4.6. Other compounds

Some compounds extracted from herbals have difficulty passing through the BBB due to their high toxicity. Triptolide, an immunosuppressive and anti-inflammatory agent extracted from the Chinese herbal medicine Tripterygium wilfordii, has shown potential neuroprotective effects associated with AD treatment. However, its clinical application to AD patients has been hindered due to high toxicity. Jia et al. (2021) reported a multicoated PLGA nanoparticle triptolide surface coated with chitosan hydrochloride, Tween-80, PEG20000, and a borneol/menthol eutectic mixture. The preparation significantly reduced cytotoxicity and inhibited apoptosis of PC12 cells induced by Aβ_1-42_. Andrographolide, the main compound from the Asian medicinal plant *Andrographis paniculata*, has been used as an alternative treatment for AD patients due to its antioxidant, neuroprotective, and anti-inflammatory properties (Bilia et al., 2019). Andrographolide has been reported to have difficulty crossing the BBB, which limits its value for treating neurological disorders. Nanoparticle-loaded andrographolide significantly improved its BBB penetration. Several other herbal extracts, such as rhynchophylline (from Uncaria) (Xu et al., 2020), ferulic acid (from Calamus) (Picone et al., 2009; Saini et al., 2021), and piperine (from piperine) (Elnaggar et al., 2015) have also been loaded into nanoparticles to increase accumulation in the brain. These data suggest that nanoparticle-loaded herbal monomeric compounds can improve bioavailability by increasing BBB permeability, alleviating toxicity, and modulating biodistribution.

## 5. Prospect

Alzheimer’s disease is a refractory neurological disease whose main pathological features are Aβ deposition and tau hyperphosphorylation in brain tissue. Drug development is mostly directed toward treating these two pathologies. Anti-AD drugs have limitations such as poor absorption, limited bioavailability, peripheral side effects, and limited BBB penetration. Furthermore, these drugs only provide symptomatic relief and do not reverse disease progression. Since 2003, few drugs have been approved by the U.S. FDA to treat AD patients. According to recent research, artificial intelligence-enabled nanomedicine approaches may help identify the optimal combination of nanomedicines for treating neurodegenerative diseases, including AD.

As a promising strategy, nanoparticles can reduce enzymatic degradation, endothelial cell clearance, and peripheral side effects while improving targeting and bioavailability and helping to overcome BBB barriers. Therefore, it is important to further develop NPs with high BBB penetrability to encapsulate natural herbs with potent anti-inflammatory and anti-apoptotic effects. In some cases, the properties of nanoparticles may be incompatible with drug binding, drug delivery, crossing the BBB, localization, and drug release. In the future, it is also necessary to design multi-component and multi-functional nanoparticles by integrating nanoparticles with different sizes, structures, and functions, and to maximize their superior properties.

Nevertheless, the clinical conversion of nanomedicines still faces many challenges. Firstly, safety is a key challenge for the clinical conversion of nanomaterials. Although some studies claim that the toxicity of natural drug-loaded nanoparticles is largely reduced, this finding is limited to preclinical data. Further studies are needed to measure the toxicity of drug-loaded nanoparticles, including their potential to cause neuroinflammation, excitotoxicity, DNA damage, and allergic reactions. A better understanding of these mechanisms can guide us in optimizing formulas, reducing or replacing adverse ingredients, and thereby reducing the risk of adverse events. Furthermore, it should be noted that while functionalized nanoparticles represent successful drug targeting, their nanoscale structure and large surface area may lead to limited particle aggregation and drug loading. Therefore, more sophisticated nanostructures need to be developed.

## 6. Conclusion

Alzheimer’s disease is one of the most dangerous diseases that threaten the physical and mental health of the elderly. As the world’s human population ages, the number of elderly individuals is increasing. However, existing treatment strategies are far from meeting therapeutic needs. Natural herbal medicines, especially Chinese herbal medicines, have received considerable attention. Their clinical use is limited due to several deficiencies. In addition, traditional drugs used for treating AD sometimes fail to reach the cells that need treatment. Nanotechnology plays a key role in enabling the treatment of many diseases, including AD. Nanoparticle-loaded compounds can effectively penetrate brain cells and treat diseased sites. Repurposing existing drugs or loading effective natural products using nanotechnology could not only greatly improve efficiency but also increase our confidence in curing AD.

## Author contributions

HL and JF: Conceptualization. NL: Writing—review & editing. NL and JR: Resources. HL: Visualization. JF: Supervision.
